# Sequelae of Constipation, Including Auto-intoxication

**Published:** 1911-12

**Authors:** Alfred J. Zobel

**Affiliations:** San Francisco, Cal.


### Abstracts and Selections.

### Sequelae of Constipation, Including Auto-intoxication.

BY ALFRED J. ZOBEL, M. D., SAN FRANCISCO, CAL.

In this paper the writer mentions many of those conditions which seem to have their origin in chronic constipation with auto-intoxication. He states that experimental evidence has not as yet demonstrated that they actually do so, but close observation and clinical experience tend strongly to confirm the theory.

He writes that while all constipated individuals do not necessarily suffer from those symptoms ascribed to auto-intoxication, yet in his experience most patients with auto-toxic symptoms are constipated. This may be without their knowledge, and they often deny in good faith that they are so; but proctoscopic examination generally proves the sigmoid and rectum to be loaded with fecal matter.

A report is given of the proctoscopic observations made on a number of cases of hypertrophic arthritis. In almost every instance the lower bowel was found filled with a fecal mass, although most of the patients positively stated that they had had an evacuation within an hour or two previous to the time of examination. Thorough colonic flushings invariably brought about relief from pain, and in time marked improvement in their general condition.

These observations are in line with the theory advanced by various authors that arthritis deformans may be due to intestinal auto-intoxication.

Mention is made of the various muscular, arthritic and neuralgic pains caused by absorption of toxins from the bowel. These are often misunderstood and treatment instituted for rheumatism.

Congestion, irritation and various disturbances, both functional and organic, of the uterus, tubes and ovaries in the female; the vesicles, urethra and prostate in the male, and the bladder in both, may result from chronic constipation. This is due both to the proximity of these organs to the lower bowel and to their close physiological relationship.

It is noted that albuminuria may arise from intestinal stasis, and mention is made of the opinion advanced by various clinicians that a nephritis may even be caused thereby.

The role of constipation with auto-intoxication as causal factors of epilepsy, neurasthenia and various mental conditions, as claimed by certain well known and competent observers, is stated here without comment.

The influence of these conditions on the heart, blood-vessels and the blood, and its effects on the eye, ear, nose and throat are dilated on in this paper, and in support of these statements quotations are culled from the literature that has appeared on this subject during the past five years.

The writer further briefly mentions a few more of those conditions that are supposed to arise from chronic constipation with auto-intoxication, and concludes by agreeing with the trite observation of Boardman Reed that, "when we except the exanthems, malaria, syphilis, tuberculosis and the diseases caused by traumatisms, by metallic poisons, and by a few other toxic agents or infections from without, practically all the remaining maladies which afflict us and cut short our lives, are now directly or indirectly traceable to auto-intoxication."—Proceedings American Proctologic Society.

---

PTERYGIUM REMOVER.—Take it off the patient's eye by touching it thoroughly with a wooden toothpick dipped in specific tincture of Lloyd's thuja occidentalis every other day. It will certainly do the work, without the use of the knife or scissors.—*Medical Brief.*

